# Comparison of electrochemical skin conductance and vibration perception threshold measurement in the detection of early diabetic neuropathy

**DOI:** 10.1371/journal.pone.0183973

**Published:** 2017-09-07

**Authors:** Amit Goel, Channabasappa Shivaprasad, Anish Kolly, Vijaya Sarathi H. A., Sridevi Atluri

**Affiliations:** Department of Endocrinology, Vydehi Institute of Medical Sciences and Research Centre, Bangalore, Karnataka, India; Weill Cornell Medical College in Qatar, QATAR

## Abstract

The early diagnosis of diabetic peripheral neuropathy (DPN) is challenging. Sudomotor dysfunction is one of the earliest detectable abnormalities in DPN. The present study aimed to determine the diagnostic performance of the electrochemical skin conductance (ESC) test in detecting early DPN, compared with the vibration perception threshold (VPT) test and diabetic neuropathy symptom (DNS) score, using the modified neuropathy disability score (NDS) as the reference standard. Five hundred and twenty-three patients with type 2 diabetes underwent an NDS-based clinical assessment for neuropathy. Participants were classified into the DPN and non-DPN groups based on the NDS (≥ 6). Both groups were evaluated further using the DNS, and VPT and ESC testing. A receiver-operator characteristic (ROC) curve analysis was performed to compare the efficacy of ESC measurements with those of DNS and VPT testing in detecting DPN. The DPN group (n = 110, 21%) had significantly higher HbA1c levels and longer diabetes durations compared with the non-DPN group (n = 413). The sensitivity of feet ESC < 60 μS, VPT testing, and DNS in detecting DPN were 85%, 72%, and 52%, respectively. The specificity of feet ESC, VPT, and DNS in detecting DPN were 85%, 90% and 60% respectively. The areas under the curves of the ROC plots for feet ESC, VPT testing, and DNS were 0.88, 0.84, and 0.6, respectively. A significant inverse linear relationship was noted between VPT and feet ESC (r = -0.45, p = <0.0001). The odds ratios for having DPN, based on the mean feet ESC testing < 60 μS, VPT testing > 15 V, and DNS ≥ 1, were 16.4, 10.9 and 1.8, respectively. ESC measurement is an objective and sensitive technique for the early detection of DPN. Feet ESC measurement was superior to VPT testing for identifying patients with early DPN.

## Introduction

Diabetic peripheral neuropathy (DPN) is a frequent complication of type 2 diabetes (T2DM), with a reported prevalence of more than 50% in long-standing cases [1]. Distal symmetric polyneuropathy, the most common form of DPN, follows a fiber length-dependent pattern and is associated with increased risks of foot deformities, ulceration, gangrene, and amputation. In the early stages, up to 50% of patients with neuropathy are asymptomatic, leading to a late diagnosis [2–4], which results in increased morbidity and mortality, and contributes to economic burden [5, 6]. Early diagnosis ensures the prompt initiation of intensive diabetes control and heightens focus on the prevention of long-term sequelae [7].

Various clinical scoring systems and bedside tests have been employed for DPN detection [8, 9]. However, the commonly used tests tend to diagnose the condition only when it is well-established and preventive measures cannot be implemented [10]. Furthermore, many of these tests are subjective, tedious, and time-consuming. Even the results of nerve conduction studies, which have been recommended for the diagnostic confirmation of DPN, can be normal in the early stages due to the preferential involvement of small unmyelinated C-fibers [11]. Sweat glands are innervated by sympathetic unmyelinated C-fibers, and sudomotor dysfunction is one of the earliest detectable abnormalities in DPN [12].

Electrochemical skin conductance (ESC) measurement using Sudoscan is an automated and objective tool for the assessment of sweat gland function. It is also non-invasive, rapid and easy to perform [12]. Vibration perception threshold (VPT) testing is a simple and commonly used point of care technique for DPN diagnosis [13]. However, its utility in the detection of early DPN compared with ESC measurement is unknown. The diabetic neuropathy symptom (DNS) score is a common screening measure for DPN. [14] However, there are sparse data on the diagnostic performance of DNS scoring for the diagnosis of early DPN, where a significant proportion of patients are asymptomatic. Furthermore, the DNS score has not been compared with objective diagnostic techniques such as VPT and ESC testing. The objective of the present study, conducted in a large cohort of Indian patients with T2DM, was to evaluate the sensitivity and specificity of ESC measurements, compared with VPT testing and the DNS, for the detection of early DPN, using a modified neuropathy disability score (NDS) as a reference standard.

## Materials and methods

### Study design

The present cross-sectional study was conducted at the Vydehi Institute of Medical Sciences and Research Centre, Bangalore, India between June and December 2016. Verbal informed consent was obtained from all the participants, and the study was approved by the Vydehi Institutional Ethics Committee, Bangalore,India. The study population consisted of 523 patients with T2DM. All consecutive patients who attended the diabetes clinic during the study period and consented for the study were recruited. Patients < 18 or > 65 years of age, those with peripheral vascular disease, chronic alcohol consumption, an active foot ulcer, or patients on drugs that could affect sweat gland activity were excluded. Patients with secondary causes of DPN such as rheumatological conditions, alcoholic polyneuropathy, untreated hypothyroidism, and hereditary neuropathy were excluded. Patients previously diagnosed with or on treatment for DPN and those with advanced stages of DPN such as foot ulcer, Charcot’s arthropathy, or amputation were also excluded. All the participants received a comprehensive neurological examination comprising five components: DNS, modified NDS, sensory function of the upper and lower extremities (pin prick, light touch, vibration, and joint position sense), bilateral reflexes, and muscle weakness evaluation. VPT and ESC measurement were performed in all the participants. Biochemical evaluations included fasting plasma glucose, glycated hemoglobin (HbA1c), complete blood count, serum creatinine, fasting lipid profile, and the urine albumin/creatinine ratio. Fundoscopy was performed in all patients. Patients were classified as having DPN based on the NDS, with an NDS ≥ 6 considered positive for DPN.

### Neurological examinations

#### DNS

The 4-item DNS is a validated and easy-to-perform symptom score for the diagnosis of DPN [14, 15]. The maximum score for DNS is 4 points, and a score of ≥ 1 suggests an abnormality.

#### Modified NDS

The modified NDS, as described by Young et al., is a validated test for the detection of DPN [16]. The minimum acceptable criteria for a diagnosis of peripheral neuropathy are moderate signs (NDS ≥ 6) with or without symptoms or moderate symptoms with at least mild signs of neuropathy (NDS ≥ 3) [16]. For the purpose of this study, DPN was defined as a modified NDS score ≥ 6. NDS was considered a reference standard when evaluating the efficacy of DNS, VPT testing, and feet ESC measurements for detecting DPN.

#### VPT

The VPT was assessed using a biothesiometer (Dhansai Lab, Mumbai, India) at 7 different body sites including the great toe, first metatarsal, third metatarsal, fifth metatarsal, medial arch, heel, and dorsum on both feet, in a graduated manner from 0 volts upwards. Patients were asked to give a verbal response once they could feel the vibration. A mean value of > 15 volts was considered abnormal [17].

#### ESC testing

Sudoscan (Impeto Medical, Paris), a device for precise evaluation of sudomotor function, was used to estimate the ESC values. The ESC test is based on an electrochemical reaction, that occurs between the chloride ions in the sweat and plate electrodes (stainless-steel), on which the patients’ feet and hands are placed. A low-voltage current (< 4 V) applied through the stainless-steel electrodes leads to the attraction of chloride ions present in the heavily concentrated sweat glands of the palms and soles [18–20]. ESC measurement is generated as a ratio of the derivative current to that over the applied low-voltage power and is expressed in micro Siemens (μS). Participants were instructed to place their hands and feet on the stainless-steel electrodes that were connected to a computer for data recording. The test lasted < 3 minutes for each participant. An alcohol-free disinfectant swab was used to clean the plate electrodes after each participant was tested to avoid potential artifacts. Sudomotor dysfunction was defined as a mean feet ESC < 60 μS.

### Biochemical investigations

Diabetes was defined according to the criteria of the American Diabetes Association. Fasting samples for plasma glucose, glycated hemoglobin, and lipids were collected and analyzed using a fully automated Beckman Coulter DXC 860i auto analyzer (Beckman Coulter, California, USA).

### Statistical analyses

Data are presented as mean ± SD for continuous variables, and percentages for categorical variables. These analyses were conducted using SAS version 9.4 software. The DPN group was defined as the participants with an NDS score ≥ 6; whereas those with an NDS score < 6 were classified as the non-DPN group. The Chi-squared and ANOVA tests were used to compare categorical and continuous variables, between the groups. Pearson’s correlation coefficient and scatter plots were utilized to assess the strength of a linear relationship between the feet ESC and VPT.

Logistic regression analysis was used to evaluate the relationship between VPT, feet ESC, DNS scores, and DPN, using the NDS test as the gold standard. A non-adjusted model and a model adjusted according to demographic and lifestyle factors (age, sex, body mass index, smoking habits, alcohol consumption, and physical activity), and on diabetes and other complications (diabetes duration, treatment, and retinopathy), were estimated.

To evaluate the performance of the three testing methods detect DPN, receiver operating characteristics curves and the respective areas-under-the-curve (AUC), and sensitivity and specificity calculations were used. A P-value < 0.05 was considered significant.

## Results

A total of 523 T2DM patients were enrolled for this study. The mean age was 49.4 ± 11.8 years and the male to female ratio was 2.7:1. The mean duration of diabetes was 4.4 ± 3.6 years and the body mass index was 24.2 ± 4.7 kg/m^2^. The mean FPG and HbA1c were 176.7 ± 61.5 mg/dl and 7.9 ± 1.6%, respectively. Diabetic retinopathy was present in 9.8% of the patients. Based on the status of the NDS, patients were divided into 2 groups–DPN group (n = 110) and non-DPN group (n = 413) [**Table 1**].

**Table 1:** Baseline characteristics of the study population according to the presence or absence of DPN.

Variables	Study group	Neuropathy according to NDS		* *
		Non-DPN	DPN	P-value*
N (%)	523	413	110	
Age (years)	49.4 ± 11.8	48.1 ± 11.4	54.4 ± 11.9	***< 0*.*0001***
Male, n (%)	385 (73.6)	298 (72.2)	87 (79.1)	*0*.*14244*
BMI (kg/m^2^)	24.2 ± 4.7	24.3 ± 4.6	23.9 ± 4.9	*0*.*51624*
Diabetes duration (years)	4.4 ± 3.6	3.4 ± 2.9	8.3 ± 3.5	***< 0*.*0001***
Smoking, n (%)	101 (19.3)	72 (17.4)	29 (26.4)	***0*.*03499***
Alcohol consumption, n (%)	46 (8.8)	31 (7.5)	15 (13.6)	***0*.*04367***
Physical activity, n (%)	267 (51.1)	215 (52.1)	52 (47.3)	*0*.*37228*
Retinopathy, n (%)	51 (9.8)	23 (5.6)	28 (25.5)	***< 0*.*0001***
DNS ≥ 1, n (%)	225 (43.0)	166 (40.2)	59 (53.6)	***0*.*01139***
NDS	1.6 ± 2.7	0.3 ± 1.1	6.5 ± 0.9	***< 0*.*0001***
NDS ≥ 6, n (%)	110 (21.0)			
VPT (volts)	13.7 ± 6.3	12.2 ± 4.2	19.4 ± 9.2	***< 0*.*0001***
VPT > 15 volts, n (%)	119 (22.8)	40 (9.7)	79 (71.8)	***< 0*.*0001***
Foot deformities, n (%)	8 (1.5)	1 (0.2)	7 (6.4)	***< 0*.*0001***
Loss of hair, n (%)	65 (12.4)	44 (10.7)	21 (19.1)	***0*.*01715***
Dry skin, n (%)	54 (10.3)	37 (9.0)	17 (15.5)	***0*.*04664***
Corn or callous, n (%)	27 (5.2)	22 (5.3)	5 (4.5)	*0*.*74205*
HbA1c (%)	7.9 ± 1.6	7.7 ± 1.4	8.8 ± 1.9	***< 0*.*0001***
FPG (mg/dl)	176.7 ± 61.5	174.8 ± 60.0	184.1 ± 66.7	*0*.*15851*
PPG (mg/dl)	247.7 ± 84.5	246.7 ± 82.7	251.5 ± 91.1	*0*.*60134*
Total cholesterol (mg/dl)	191.3 ± 47.6	190.2 ± 47.4	195.7 ± 48.6	*0*.*28214*
Triglycerides (mg/dl)	142.4 ± 103.2	139.8 ± 102.9	152.2 ± 104.4	*0*.*26226*
HDL (mg/dl)	43.2 ± 9.2	43.1 ± 9.1	43.7 ± 9.4	*0*.*54385*
LDL (mg/dl)	99.4 ± 33.8	97.6 ± 33.3	106.3 ± 34.9	***0*.*01618***
Vitamin B12 (pg/ml)	433.6 ± 186.2	434.0 ± 192.4	432.0 ± 161.3	*0*.*91759*
TSH (uIU/ml)	2.4 ± 1.2	2.4 ± 1.2	2.4 ± 1.0	*0*.*78859*
Urine protein/creatinine ratio	0.2 ± 0.5	0.2 ± 0.4	0.3 ± 0.6	*0*.*08528*
Feet ESC (μS)	63.9 ± 18.2	69.3 ± 13.8	43.7 ± 18.7	***< 0*.*0001***
Feet ESC < 60, n (%)	153 (29.3)	60 (14.5)	93 (84.5)	***< 0*.*0001***
Abnormal hands or feet ESC, n (%)	257 (49.1)	157 (38.0)	100 (90.9)	***< 0*.*0001***

N (%) for categorical variables, mean (SD) for continuous variables.

*P-value < 0.05 considered significant.

BMI–body mass index, DNS–Diabetic neuropathy symptom score, NDS–modified neuropathy disability score, VPT–vibration perception threshold, FPG–fasting plasma glucose, PPG–post prandial glucose, HbA1c –glycated hemoglobin, HDL–high density lipoprotein, LDL–low density lipoprotein, TSH–thyroid stimulating hormone, ESC–electrochemical skin conductance

**Fig 1:**
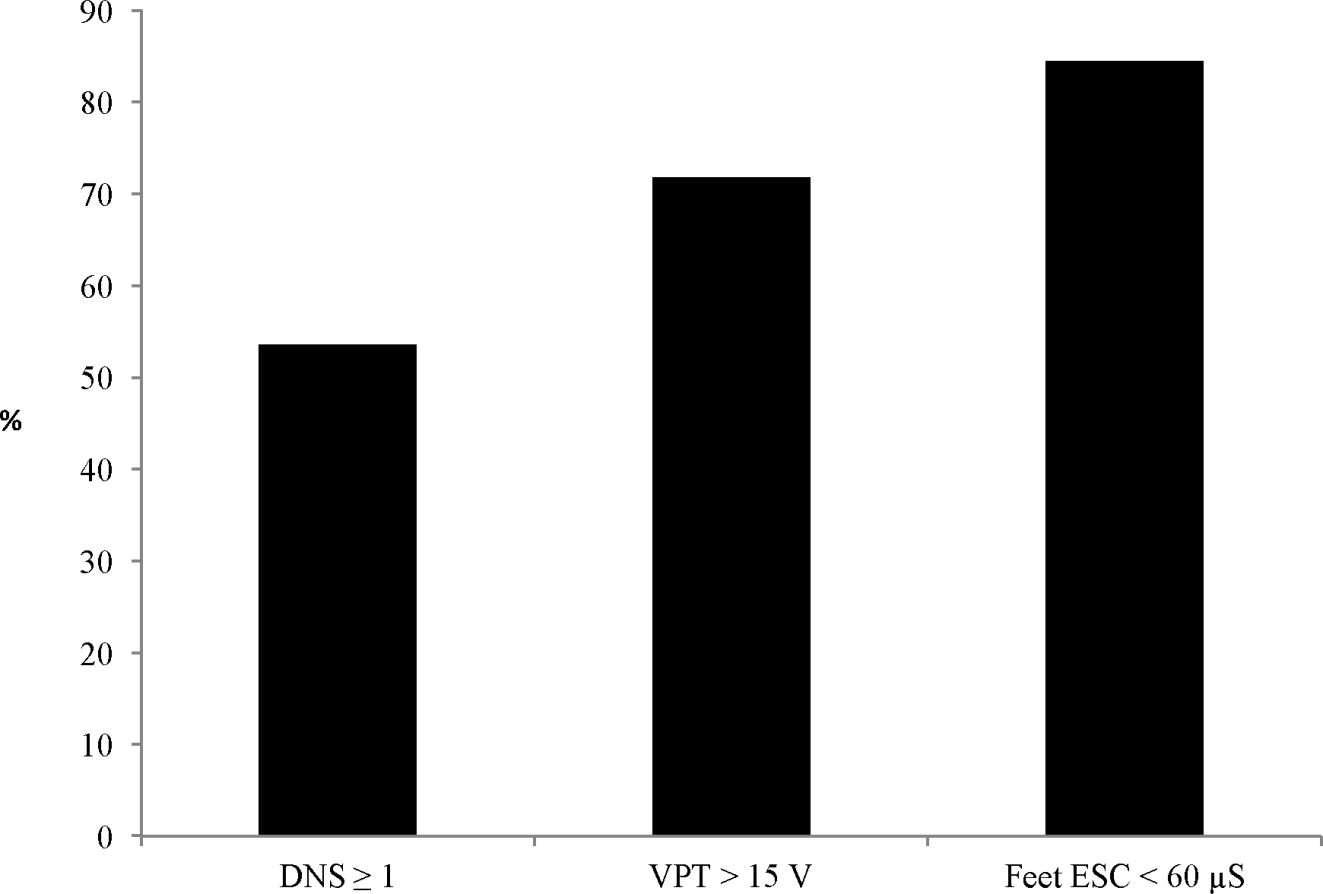
Percentages of abnormal DNS scores, and VPT or feet ESC measurements in patients with DPN, as defined by NDS ≥ 6 (n = 110). DNS—diabetic neuropathy symptom score, n = 59 (54%). VPT- vibration perception threshold, n = 79 (72%). Feet ESC- feet electrochemical skin conductance, n = 93 (84%).

Based on the NDS score, patients were divided into the DPN (NDS ≥ 6) (n = 110) and non-DPN (NDS< 6) (n = 413) groups. Patients in the DPN group (n = 110, 21%) were significantly older and had a longer mean diabetes duration compared with those in the non-DPN group (n = 413, 79%). In the DPN group, the FPG, HbA1c, and mean VPT scores were significantly higher, and the mean ESC score was significantly lower, compared with the non-DPN group. In patients with DPN (n = 110), the prevalence of abnormal DNS scores, and VPT and feet ESC measurements were 59 (54%), 79 (72%), and 93 (85%), respectively (**Fig 1)**.

The diagnostic performance of abnormal DNS scores, and VPT and feet ESC measurements for DPN detection considering NDS as the reference standard, are provided in **Table 2**.

**Table 2:** Diagnostic performance of the feet ESC, VPT, and DNS in detecting DPN.

	Cutoff	Sensitivity	Specificity	PPV	NPV	AUC
DNS	≥ 1	52%	60%	25%	83%	0.60
VPT	> 15 V	72%	90%	66%	92%	0.84
Feet ESC	< 60 μS	85%	85%	61%	95%	0.88

DNS–diabetic neuropathy symptom score, VPT–vibration perception threshold, Feet ESC–feet electrochemical skin conductance, PPV–positive predictive value, NPV–negative predictive value, AUC–area under curve

**Fig 2:**
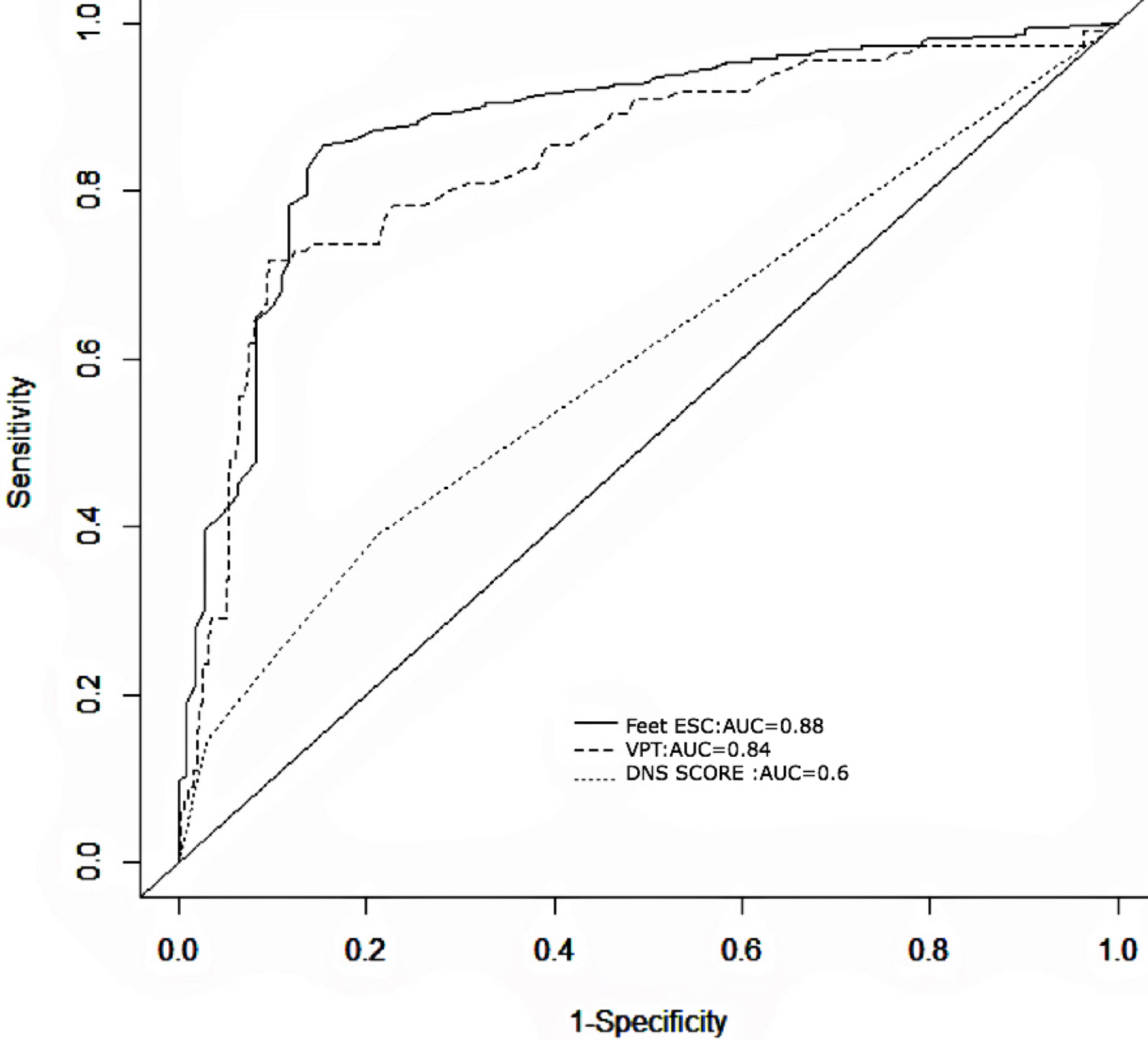
Receiver operating characteristics (ROC) curves for the DNS, feet ESC, and VPT scores, for detecting DPN, considering NDS ≥ 6 as the reference standard. DNS–diabetic neuropathy symptom score, VPT–vibration perception threshold, Feet ESC–feet electrochemical skin conductance.

The feet ESC displayed a higher sensitivity (85% vs. 72%) and an equivalent specificity (85% vs. 90%) for classifying DPN, compared with the mean VPT. The sensitivity (52%) and specificity (60%) of the DNS score were lower than both the feet ESC and VPT values. The AUCs of the ROC plots for DNS scores, VPT values, and feet ESC measurements were 0.6, 0.84, and 0.88, respectively (Fig 2).

There was a significant inverse linear relationship between the VPT and feet ESC values (r = -0.45, p < 0.0001). A significantly higher proportion of patients with retinopathy (n = 31, 61%) had an abnormal mean feet ESC (< 60 μS) when compared to patients without retinopathy (n = 122, 26%; p = 0.001). After adjustment for potential confounding variables, the odds ratios for having DPN, based on the mean feet ESC (< 60 μS), VPT >15 V, and DNS score ≥ 1, were 16.4, 10.8, and 1.8, respectively (**Table 3**).

**Table 3:** Odds ratio (95% CI) of having DPN according to the feet ESC, VPT, and DNS values.

	Unadjusted model	Adjusted model *
Feet ESC ≥ 60 μS	1	1
Feet ESC < 60 μS	32.18 (17.93–57.76)	16.37 (7.99–33.53)
*p for trend*	*< 0*.*0001*	*< 0*.*0001*
DNS < 1	1	1
DNS ≥ 1	1.72 (1.13–2.63)	1.81 (1.05–3.14)
*p for trend*	*< 0*.*0001*	*0*.*0004*
VPT ≤ 15 V	1	1
VPT > 15 V	23.76 (14.01–40.30)	10.87 (5.69–20.76)
*p for trend*	*< 0*.*0001*	*< 0*.*0001*

*model adjusted for age, sex, body mass index, diabetes duration, smoking, alcohol consumption

physical activity, retinopathy, and treatment of diabetes

## Discussion

To our knowledge, this is the largest study from the Indian sub-continent to evaluate the performance of the ESC test against other well-established methods for the detection of DPN. The study demonstrates that feet ESC <60 μS is a sensitive and specific marker for the diagnosis of DPN. The feet ESC value demonstrated a significant inverse linear relationship with the VPT value and had a superior sensitivity for DPN detection.

NDS is a validated clinical score with a high sensitivity and specificity for the diagnosis of distal symmetric polyneuropathy [21, 22]. Patients with an NDS ≥ 6 points are considered to display abnormal reactions [8, 10, 23]. Furthermore, an NDS ≥ 6 has been shown associated with an increased risk of foot ulceration [24–27], poor glycemic control, and microvascular complications [28]. Several studies have demonstrated the utility of NDS in the diagnosis of DPN [29–33]. Therefore, for the present study, the NDS was considered as the reference standard against which the diagnostic performances of the DNS score and VPT and feet ESC measurements were evaluated.

In the present study, DPN prevalence, as defined by an NDS score ≥ 6, was 21.0%, which was similar to the estimates of neuropathy prevalence found in previous studies, when matched for the T2DM duration [16, 34–35]. Young et al. noted a DPN prevalence of 20.8% in patients who had diabetes for < 5 years [16]. In a Finnish longitudinal study in similar patients, the DPN prevalence, defined by nerve conduction abnormalities, was 16.7% [34]. The DPN prevalence reported in previous studies conducted in Indian patients ranged from 26.1% to 29.2% [9, 36–38]. However, the mean diabetes duration and HbA1c were higher in those Indian patients compared with those in the present study. The lower prevalence noted in our study might also be attributed to the exclusion of patients with advanced stages of DPN. Differing prevalence estimates could also be partly attributed to the diagnostic criteria for DPN, study design, and variations in sampling methods.

The present findings demonstrate that, after the careful characterization of DPN, a feet ESC threshold < 60 μs showed excellent sensitivity and specificity for detecting DPN. The area under the ROC curve for the detection of DPN showed a significant result for feet ESC testing. These results are congruent with those from previous studies that evaluated the performance of ESC measurement against different traditional methods for diagnosing DPN [39–43]. Selvarajah et al., evaluated feet ESC (< 60 μs) testing for classifying DPN, based on the American Academy of Neurology criteria. They found sensitivity and specificity values of 87.5% and 76.2%, respectively [39], with a reported area under the ROC curve of 0.85, which was similar to that in the present study. Casellini et al. evaluated the efficacy of ESC measurement (< 60 μS) for detecting DPN, compared with 3 other traditional modalities (quantitative sensory testing, quantitative autonomic function testing, and neuropathy impairment score-lower legs). In their study, the sensitivity, specificity, and area under the ROC curve for feet ESC testing were 92%, 78%, and 0.88, respectively [40]. Sheshah et al. demonstrated that the sensitivities of feet ESC testing (< 70 μS) for detecting DPN, defined by VPT ≥ 25 V, NDS ≥ 3, and NDS ≥ 6, were 100%, 80.6%, and 80.9%, respectively [41].

In the present study, the diagnostic sensitivity and overall diagnostic performance of feet ESC were superior compared with those of VPT testing or DNS score. Multivariate logistic regression revealed that the odds ratio for DPN, based on abnormal feet ESC values (< 60 μS), was 16.4. In comparison, the odds ratios for identifying DPN, based on abnormal VPT and DNS, were 10.8 and 1.8, respectively. Previous studies have shown that there is a significant inverse linear correlation between VPT and ESC [19, 41, 44]. Using VPT as a reference test, it has been reported that the sensitivities and specificities of ESC testing for DPN classification ranged between 73–82% and 55–62%, respectively [20, 45]. However, on directly comparing VPT and ESC testing, we observed a higher sensitivity and an equivalent specificity for the latter, in detecting DPN.

A few characteristics of the study population are noteworthy. A significant proportion of patients in the DPN group were asymptomatic, as evidenced by a normal DNS. Patients with advanced stages of DPN were excluded. Furthermore, there was a low prevalence of diabetic retinopathy. In addition, the reported mean age, and the duration of T2DM and HbA1c were lower in our patients when compared with previous studies evaluating ESC [39, 41, 44]. These factors reiterate that patients in the DPN group had early-stage DPN. ESC testing evaluates the sweat nerve fibers innervated by the sympathetic unmyelinated C-fibers, which are lost in the early stages of DPN [20]. In contrast, the nerve fibers mediating vibration sense are larger and myelinated (Aβ) and are typically involved in the later stages of neuropathy [46, 47]. Therefore, the lower sensitivity of VPT testing observed in the present study could be attributed to the later involvement of these fibers in DPN. It has also been noted that the selective loss of vibration sense is rare in the early stages of neuropathy [47, 48]. Impaired vibration perception has previously been shown to be strongly associated with diabetic foot ulceration [49]. The present findings suggest that ESC testing might be better for the early detection of DPN compared with VPT testing and that VPT testing might be more useful for detecting established neuropathy and the risk of foot ulceration.

Compared with VPT and ESC testing, the DNS score had poor sensitivity and specificity for detecting DPN. Although a few studies have observed high sensitivity of DNS in screening for DPN, they might be less reliable due to their subjectivity [50]. The poor reproducibility of symptom scores by different observers has been noted [51]. The poor specificity of DNS score in accurately detecting DPN has also been reported [9]. In detecting early neuropathy, where a significant proportion of patients are asymptomatic, DNS scoring might not be useful.

We did not compare the ESC measurement with other diagnostic tests for small fiber neuropathy, such as the intra-epidermal nerve fiber density or quantitative sudomotor axon reflex test. The present study was cross-sectional in design and follow-up data are required to complement the results. Although we did not evaluate the performance of the ESC measurement to screen for cardiac autonomic neuropathy, it has been validated previously in Indian diabetic patients [20]. Further studies involving larger cohorts, divergent stages of neuropathy, and different age groups are required to endorse these findings. In addition, interventional studies to evaluate the effectiveness of this technique, as a tool for early detection of small nerve-fiber dysfunction and peripheral autonomic neuropathy are needed.

In conclusion, DPN is often asymptomatic, and there is a need for simple, accurate, and quantitative methods for early diagnosis. ESC measurement is a sensitive, non-invasive, and objective test for the early detection of DPN. It allows for the early identification of patients with small unmyelinated sympathetic nerve fiber dysfunction and can be used as a screening test for peripheral neuropathy.

## Supporting information

S1 Dataset Original dataset. (XLSX)
